# The claustrum of the ferret: afferent and efferent connections to lower and higher order visual cortical areas

**DOI:** 10.3389/fnsys.2014.00031

**Published:** 2014-02-28

**Authors:** Nina Patzke, Giorgio M. Innocenti, Paul R. Manger

**Affiliations:** ^1^School of Anatomical Sciences, Faculty of Health Sciences, University of the WitwatersrandJohannesburg, South Africa; ^2^Department of Neuroscience, Karolinska InstitutetStockholm, Sweden; ^3^Brain and Mind Institute, École Polytechnique Fédérale de LausanneLausanne, Switzerland

**Keywords:** cerebral cortex, claustrum, connections, ferret, visual cortex

## Abstract

The claustrum, a subcortical telencephalic structure, is known to be reciprocally interconnected to almost all cortical regions; however, a systematic analysis of claustrocortical connectivity with physiologically identified lower and higher order visual cortical areas has not been undertaken. In the current study we used biotinylated dextran amine to trace the connections of the ferret claustrum with lower (occipital areas 17, 18, 19 and 21) and higher (parietal and temporal areas posterior parietal caudal visual area (PPc), posterior parietal rostral visual area (PPr), 20a, 20b, anterior ectosylvian visual area (AEV)) order visual cortical areas. No connections between the claustrum and area 17 were observed. Occipital visual areas 18, 19 and 21 revealed a reciprocal connectivity mainly to the caudal part of the claustrum. After injection into parietal areas PPc and PPr labeled neurons and terminals were found throughout almost the entire rostrocaudal extent of the dorsal claustrum. Area 20b revealed reciprocal connections mainly to the caudal-ventral claustrum, although some labeled neurons and terminals were observed in the dorso-central claustrum. No projection from the claustrum to areas AEV and 20a could be observed, though projections from AEV and 20a to the claustrum were found. Only injections placed in areas PPr and AEV resulted in anterogradely labeled terminals in the contralateral claustrum. Our results suggest that lower order visual areas have clearly defined connectivity zones located in the caudal claustrum, whereas higher order visual areas, even if not sending and/or receiving projections from the entire claustrum, show a more widespread connectivity.

## Introduction

The claustrum is a thin subcortical unlaminated telencephalic structure located between the dorsal striatopallidal complex, specifically the putamen, and the insular cortex, or claustrocortex in mammals that lack the insular formation and a sylvian fissure. A precise definition of the anatomical location of the claustrum within the mammalian brain has been recently advanced based on proteomic studies, and indicates that the claustrum is surrounded by layer VI neurons and is connected to the cerebral cortex, but it does not connect to sub-cortical structures (Mathur et al., 2009). The claustrum appears to be present in all mammals (Jakubowska-Sadowska et al., 1998; Kowianski et al., 1999), but evidence for its existence in all monotremes is equivocal (Butler et al., 2002; Ashwell et al., 2004). Various anatomical tract-tracing experiments, mostly in cats and monkeys, but also in other species, have demonstrated that the claustrum is reciprocally interconnected to almost all cortical regions. Those cortical regions for which claustrocortical connectivity has been observed include: visual cortex, parieto-occipital and posterior parietal cortex, temporal and temporopolar cortex, motor and premotor cortex, prefrontal cortex, cingulate cortex, the frontoparietal operculum, somatosensory cortex, prepiriform olfactory cortex and the entorhinal cortex (for details see Tanné-Gariépy et al., 2002; Edelstein and Denaro, 2004; Crick and Koch, 2005). Tractographic anatomical studies in humans have demonstrated claustral projections to the superior frontal, precentral, postcentral, posterior parietal, orbitofrontal, prefrontal, temporal and occipital cortical regions (Fernández-Miranda et al., 2008).

The claustrocortical connections are topographically organized, forming cortical projection zones within the claustrum; however, these projection zones are not strictly separated from each other and appear to overlap. The degree of overlap, the extent and the location of the projection zones within the claustrum are species-specific (Jakubowska-Sadowska et al., 1998). Examples for such a species-specific variation are the claustral visual projection zones. For example, the claustral projections zones to areas 17 and 18 show a full overlap in rat and rabbit, but are completely separate in the cat (Jakubowska-Sadowska et al., 1998). Moreover the visual projection zones in the claustrum of rat, rabbit and primates are located in the caudoventral claustrum, whereas in the cat and guinea pig they occupy the caudodorsal claustrum (LeVay and Sherk, 1981; Jakubowska-Sadowska et al., 1998). The claustrum is mainly reciprocally connected to the ipsilateral cortex, but inconsistent data concerning the presence of a weaker contralateral projection has been reported for different species (e.g., Jakubowska-Sadowska et al., 1998).

Contrasting with the topographically organized functional/connectional subdivisions of the claustrum, immunohistochemical and Golgi studies have revealed a homogeneous architecture without any parcellation (Sherk, 1986; Reynhout and Baizer, 1999). Due to this uniform organization, it has been suggested that neuronal information processing within the claustrum may entail specialized mechanisms that allow information to travel widely within its rostral-caudal and dorsal-ventral extent (Crick and Koch, 2005). In light of the extensive connectivity of the claustrum to virtually all cortical areas, and its intra-claustral organization, Crick and Koch (2005) postulated that the claustrum may synchronize information both within and across sensory, motor and cognitive modalities, thus enabling a conscious perception of environmental stimuli.

Although many studies of claustrocortical connectivity have been conducted, there is a lot of inconsistency in the reports. Importantly, a systematic analysis of claustrocortical connectivity with established and physiologically identifiable lower and higher order visual cortical areas is missing from the literature—i.e., are systematic differences in the extent, location and connectivity type evident as one proceeds from lower order to higher order areas? In the current study we used biotinylated dextran amine (BDA) tracing to examine the connectivity of the ferret claustrum with physiologically identified lower (occipital areas 17, 18, 19 and 21; Innocenti et al., 2002; Manger et al., 2002a) and higher (parietal and temporal posterior parietal caudal visual area (PPc), posterior parietal rostral visual area (PPr), 20a, 20b, anterior ectosylvian visual area (AEV; Manger et al., 2002b, 2004, 2005)) order visual cortical areas to specifically answer the question.

## Materials and methods

**Figure 1:**
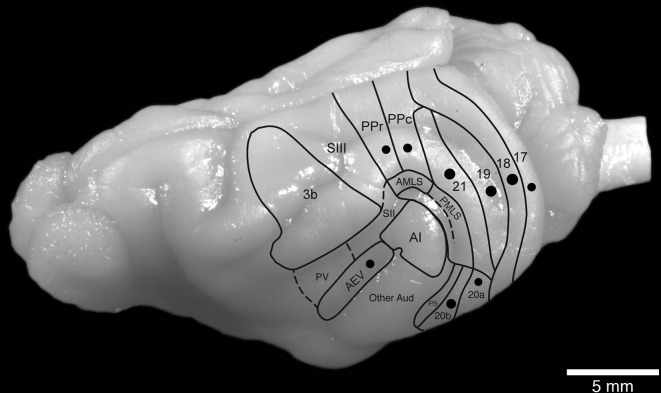
**Location and approximate size of the injection sites analyzed in the current study in relation to the known boundaries of visual cortical areas in the ferret brain**.

Nine adult female ferrets (*Mustela putorius*) weighing between 600 and 1000 g were used in the current study. All experiments were performed according to Swedish and European Community guidelines for the care and use of animals in scientific experiments. The animals were initially anesthetized with i.m. doses of ketamine hydrochloride (Ketalar, 10 mg/kg) and medetomidin hydrochloride (Domitor, 0.08 mg/kg), supplemented with atropine sulfate (0.15 mg/kg) and placed in a stereotaxic frame. A mixture of 1% isoflurane in a 1:1 nitrous oxide and oxygen mixture was delivered through a mask while the animal maintained its own respiration. Anesthetic level was monitored using the eye blink and withdrawal reflexes, in combination with measurement of the heart rate. The visual cortex was exposed under aseptic conditions and in each animal a number (less than 20) of electrophysiological recordings were taken to ensure placement of the tracer within a specific cortical area near the representation of the horizontal meridian (Manger et al., 2002a,b, 2004, 2005). Approximately 500 nl of tracer (biotinylated dextran amine, BDA 10 k, 5% in 0.1 M phosphate buffer; Molecular Probes) was delivered at each injection site (one site per animal) through a Hamilton microsyringe (Figure 1).

After completion of the injection, a soft contact lens was cut to fit over the exposed cortex, the retracted dura mater pulled over the contact lens, and the excised portion of bone repositioned and held in place with dental acrylic. The temporal muscle was reattached using surgical glue and the midline incision of the skin sutured. Antibiotics were administered in all cases (Terramycin, 40 mg/kg, each day for 5 days). These animals were given a 2-week recovery period to allow for tracer transport. At the end of this period, the animals were given a lethal dose of sodium pentobarbital (80 mg/kg, i.p.) and perfused intracardially, initially with a rinse of 0.9% saline (4°C, 500 ml/kg), followed by fixation with 4% paraformaldehyde in 0.1 M phosphate buffer (4°C, 1000 ml/kg). The brain was removed from the skull and post-fixed overnight in the same solution, then transferred to a 30% sucrose solution in 0.1 M phosphate buffer (at 4°C) and allowed to equilibrate. The brains were then frozen in dry ice and sectioned on a freezing microtome in a coronal plane and a one in four series of stains made for Nissl substance (with cresyl violet), myelin (Gallyas, 1979), cytochrome oxidase (Carroll and Wong-Riley, 1984) and BDA. For visualization of BDA, the sections were incubated in 0.5% bovine serum albumin in 0.05 M Tris buffer for 1 h. This was followed by incubation in an avidin-HRP solution for 3 h. A 10 min pre-incubation in 0.2% NiNH_4_SO_4_ preceded the addition of H_2_O_2_ (200 μl/l) to this solution, at which time the sections were monitored visually for the reaction product. The reaction was stopped by placing the sections in 0.05 M Tris buffer. All sections were mounted on 0.5% gelatine coated slides, dehydrated in graded series of alcohols, cleared in xylene and coverslipped with Depex mounting medium.

The stained sections were examined at both low and high power with light microscopes to determine in which sections through the claustrum labeled cell bodies and terminals were present. Under low power stereomicroscopy, the architectonic outlines of the claustrum and adjacent regions were drawn using the Nissl, myelin and cytochrome oxidase sections with the aid of a camera lucida. The sections reacted for the anatomical tract tracer were then matched to these drawing and the locations of individual retrogradely labeled cells plotted and regions of anterogradely labeled axonal terminal demarcated. The drawings were scanned and redrawn using the Canvas 8 drawing program. Digital photomicrographs were captured using a Zeiss Axioskop and the Axiovision software. No pixilation adjustments, or manipulation of the captured images were undertaken, except for the adjustment of contrast, brightness, and levels using Adobe Photoshop 7.

## Results

**Figure 2:**
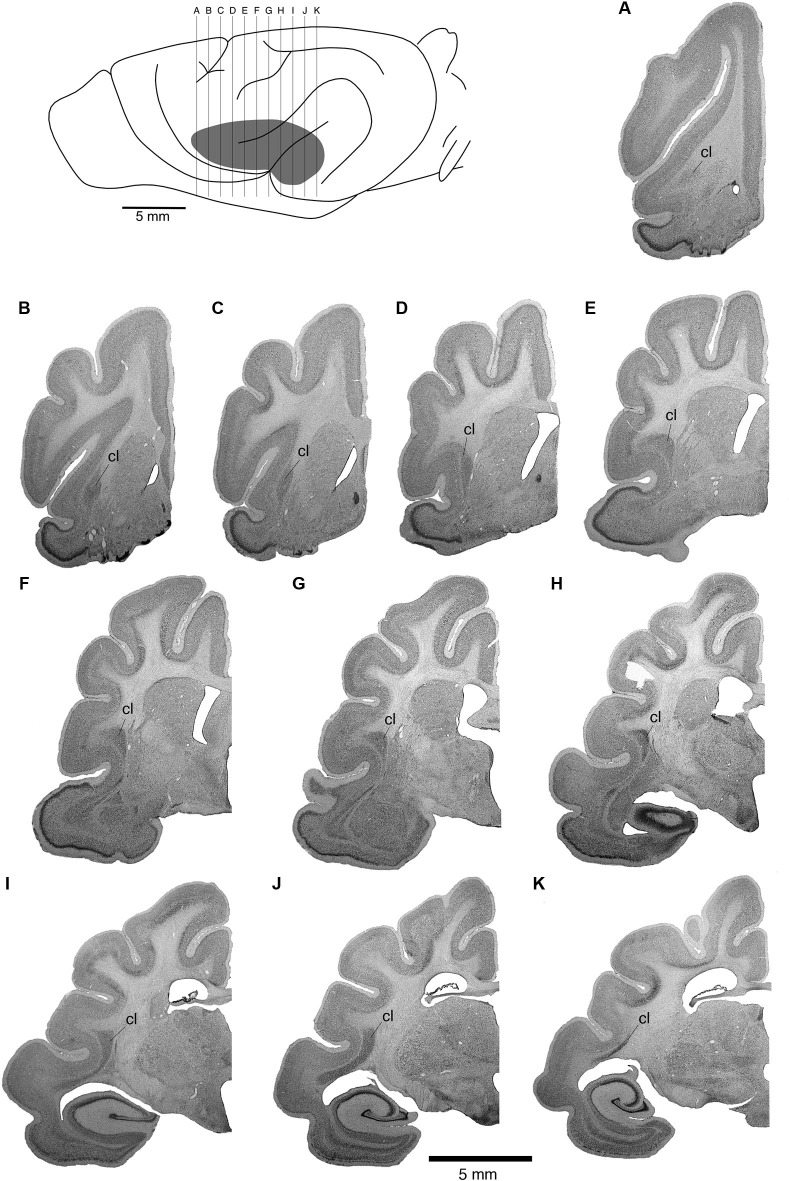
**Location of the ferret claustrum (cl)**. The drawing of the lateral view of the ferret brain demarcates the location of the claustrum below the cerebral cortex, as well as the level of section corresponding to each of the images of the rostral to caudal Nissl stained sections **A** through **K** which are approximately 1 mm apart.

The position of the claustrum and its extent was identified using Nissl, myelin and cytochrome oxidase stained sections that were made adjacent to the sections on which the BDA labeling was revealed and analyzed. The claustrum of the ferret shows the typical mammalian shape of a thin, curved, irregular sheet of gray matter, located between the putamen and the claustrocortex (Figure 2). In the rostrocaudal axis, the claustrum was found to extend from the most rostral part of globus pallidus to the rostral border of the habenular complex (Figure 2).

### Claustral connections with the lower order/occipital visual areas 17, 18, 19 and 21

**Figure 3:**
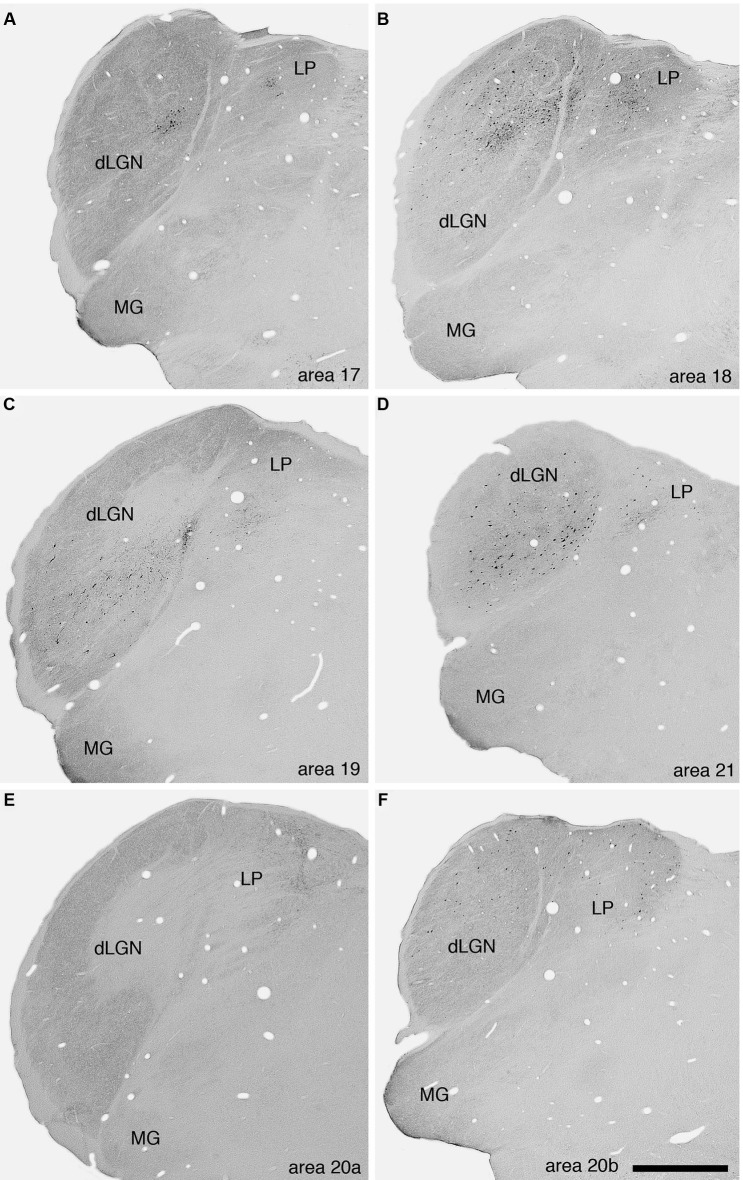
**Photomicrographs of labeled cells and axon terminals in the visual regions of the dorsal thalamus of the ferret following injection of BDA into various visual cortical areas.** **A—**area 17, **B—**area 18, **C—**area 19, **D—**area 21, **E—**area 20a, **F—**area 20b. Note the presence of labeled cells or axon terminals in the **dLGN** or the lateral posterior nucleus (**LP**) following the injections into the different cortical areas. Scale bar in **F** = 1 mm and applies to all. Dorsal to the top and medial to the right in all images. **MG—**medial geniculate body.

No BDA positive neurons or terminals were observed in the claustrum after injection of the tracer into area 17. As a control we examined the potential connections with the dorsal lateral geniculate nucleus (dLGN) and observed labeled cells (Figure 3A). This connectivity with the dLGN in the ferret following area 17 injections indicates that the lack of labeled cells in the claustrum is not a result of methodological issues. Additional examples of dorsal thalamic connectivity following injections into the different visual areas indicates that in all cases the injections were correctly placed in the cerebral cortex (Figure 3).

**Figure 4:**
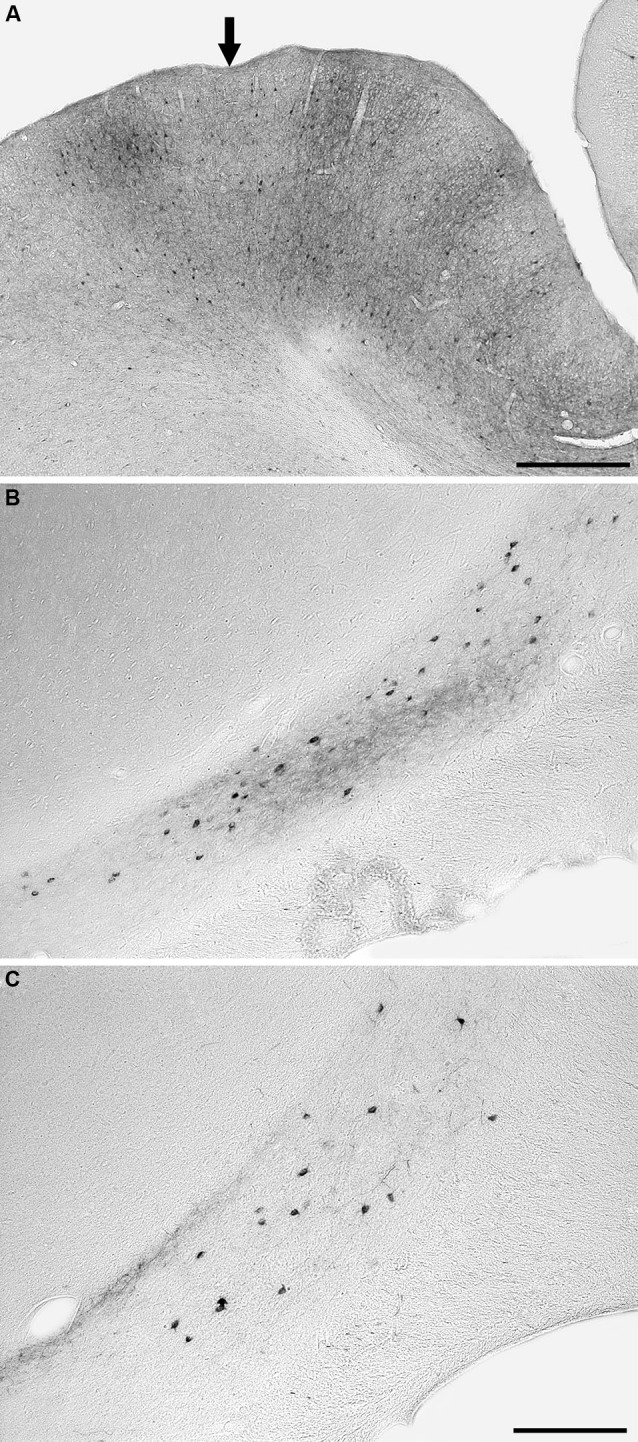
**Photomicrographs of an example injection site in area 21 (A) and labeled cells and terminals in the claustrum following injections into area 18 (B) and area 21 (C)**. Note that the injected tracer has been mostly taken up from the injection site (entry of microsyringe into cortex marked with an **arrow**) and that the injection sites did not encompass any of the underlying white matter. Following injections in both areas 18 and 21, retrogradely labeled neurons and anterogradely labeled terminals were observed, but in the depicted cases the anterograde labeling was far stronger after injection into area 18. In all images medial is to the right and dorsal to the top. The scale bar in **A** = 500 μm, the scale bar in **C** = 250 μm and applies to **B** and **C**.

**Figure 5:**
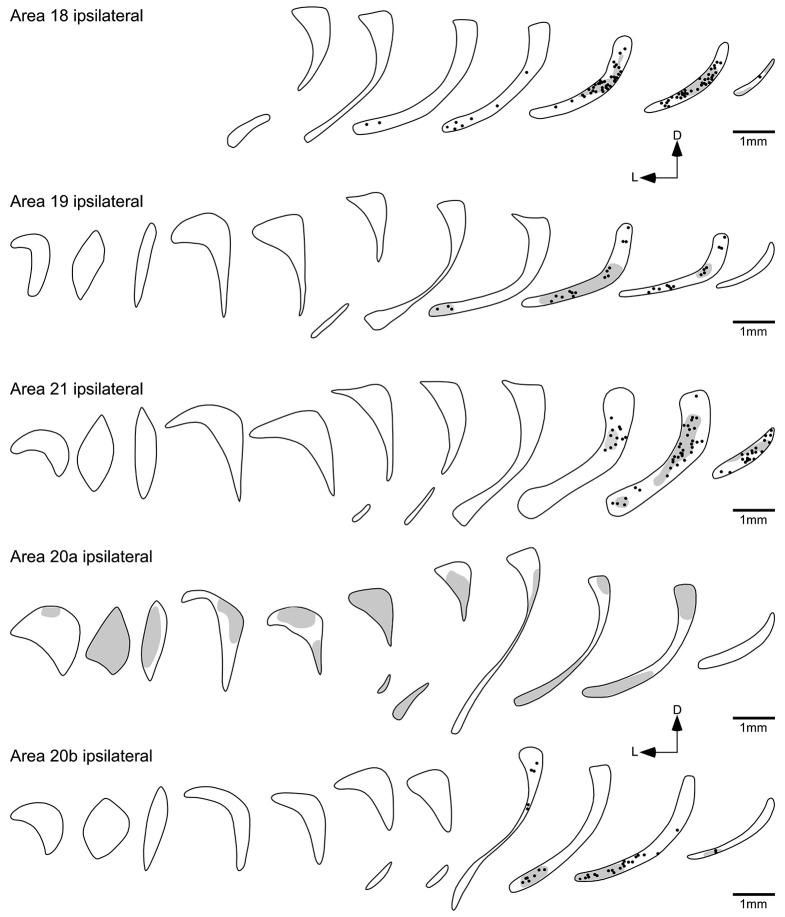
**Diagrammatic reconstructions of the ipsilateral claustral connectivity following injections of biotinylated dextran amine into cortical visual areas 18, 19, 21, 20a and 20b**. Each figurine, which are spaced approximately 1 mm apart in the rostrocaudal plane, represents a coronal section through the claustrum (see Figure 2) with the rostral sections to the left and caudal sections to the right and for each figurine medial is to the right and dorsal to the top. In each figurine, the dots represent retrogradely labeled neurons, while the shading represents regions of labeled anterograde axonal terminals. Note the caudally located reciprocal connectivity of the occipital (18, 19, 21) visual areas and also the temporal visual area 20b, while temporal visual area 20a shows a widespread anterograde connectivity to the claustrum.

Injections into visual areas 18, 19 and 21 revealed similar projection zones in the claustrum (Figures 4, 5). Retrogradely labeled neurons were exclusively found in the caudal third of the ipsilateral claustrum. In the caudal half of the projection zone labeled neurons were located throughout the whole cross section of the claustrum, with the highest cell density in the middle portion of the cross section. Injections into areas 18 and 19 revealed a few labeled neurons in a more rostral location occupying the ventral portion of the cross section. Injections into area 21 revealed the smallest projection zone in the claustrum and at the most rostral location where labeled cell were present, they were observed in the dorsal portion of the cross section. For all three visual areas the presence of anterogradely labeled axonal terminals was concurrent with the territories where retrogradely BDA labeled neurons were observed. No BDA positive cells or terminals were observed in the contralateral claustrum following injection into these cortical areas.

### Claustral connections with the posterior parietal visual areas PPc and PPr

**Figure 6:**
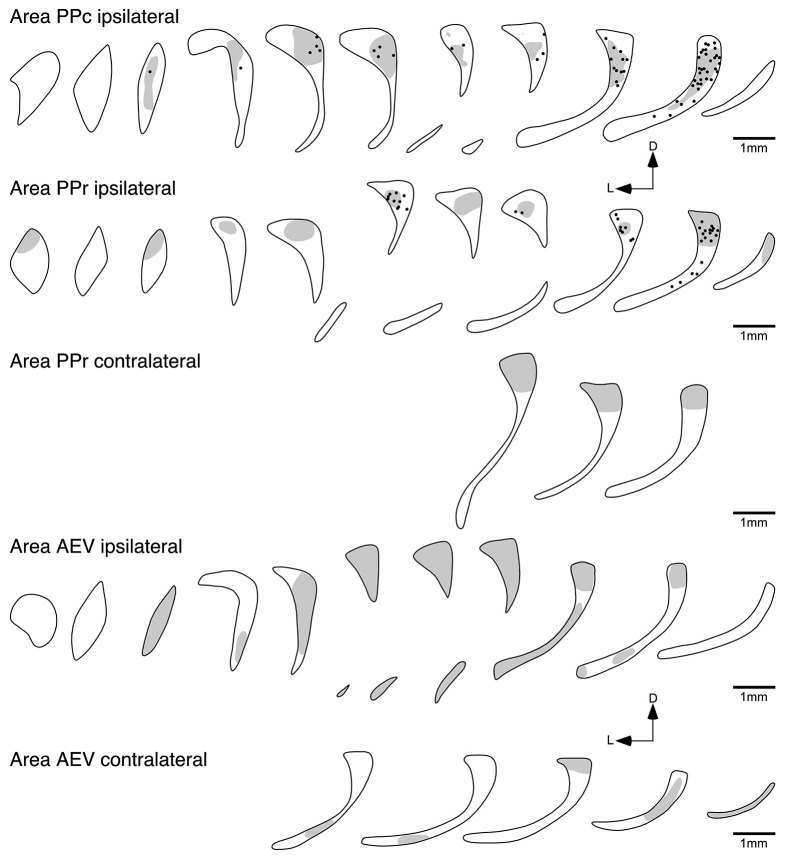
**Diagrammatic reconstructions of the ipsilateral and contralateral claustral connectivity following injections of biotinylated dextran amine into cortical visual areas PPc, PPr and AEV.** Each figurine, which are spaced approximately 1 mm apart in the rostrocaudal plane, represents a coronal section through the claustrum (see Figure 1), with the rostral sections to the left and caudal sections to the right and for each figurine medial is to the right and dorsal to the top. In each figurine, the dots represent retrogradely labeled neurons, while the shading represents regions of labeled anterograde axonal terminals. Note the widespread ipsilateral reciprocal connectivity of areas PPc and PPr, the widespread ipsilateral anterograde connectivity of area AEV, with a small amount of contralateral anterograde connectivity following injections in areas PPr and AEV.

Following injection into area PPc, BDA positive neurons were found throughout the majority of the rostrocaudal extent of the ipsilateral claustrum, and were seen to occupy the dorsal half of the cross section (Figure 6). The number of labeled cells exhibited a greater density towards the caudal pole of the claustrum; however, at the most rostral and caudal poles of the claustrum no labeled neurons were observed. Anterogradely labeled axonal terminals were coincident with the regions where retrogradely BDA labeled neurons were observed.

Injections of anatomical tract tracer into area PPr resulted in a labeling of neurons mostly in the caudal half of the ipsilateral claustrum. BDA positive neurons were observed to occupy the dorsal portion of the cross section, with the highest density of labeled cells observed in the caudal part of the claustrum; however, as with the PPc injections no labeled neurons were observed at the most caudal pole of the claustrum. The region of the claustrum in which retrogradely labeled neurons were observed also contained anterograde labeling, but this extended over greater rostrocaudal extent of the claustrum. Interestingly, anterogradely labeled terminals were observed in the dorsal caudal aspect of the contralateral claustrum following injection of BDA into area PPr (Figure 6).

### Injection into the temporal visual areas 20a, 20b and AEV

Although areas 20a and 20b are small adjacent visual areas in the temporal lobe (Manger et al., 2004), the labeling in the claustrum following cortical injection showed quite distinct and differing patterns for each area (Figure 5). The injections into 20b resulted in the labeling of cell and axonal terminals in the caudal third of the ipsilateral claustrum, mostly located in the ventral half of the claustrum; however, in the most rostral part of the projection zone labeled cells were observed in the dorsal half of the claustrum.

In contrast, no labeled cells were observed in the claustrum after injection into area 20a (Figure 5); however, anterogradely labeled axonal terminals were scattered through the entire ipsilateral claustrum. Similar to the claustral projection observed with area 20a, no labeled neurons were observed in the claustrum following injection into area AEV (Figure 6). Here again anterogradely labeled axonal terminals were observed throughout the entire ipsilateral claustrum; however, no labeling was found at the most rostral and caudal poles of the claustrum. As with area PPr, a few randomly scattered labeled axonal terminals were observed in the caudal half of the contralateral claustrum following injection into area AEV (Figure 6).

## Discussion

Claustrocortical connectivity has been the subject of several studies on different animals (see below); however, a systematic analysis of the connectivity with established and physiologically identified visual cortical areas has not been undertaken. In the current study, the interconnections of several physiologically identified lower and higher order visual areas with the claustrum was systematically examined in the ferret.

### Claustrocortical connectivity with the lower order visual area 17

No connectivity between the claustrum and area 17 was observed in the present study; however, BDA positive neurons were found in the lateral geniculate nucleus—the principal thalamic input neurons to area 17—indicating that the injection site was correctly placed. Previous studies describing claustrocortical connectivity with area 17 present inconsistent results. While some studies show no connectivity of the claustrum with area 17 in rat (Hadley and Trachtenberg, 1978; Carey and Neal, 1985), hamster (Lent, 1982) and owl monkey (Graham et al., 1979), other tracing studies show contrary results. For example, a comparative tracing study by Jakubowska-Sadowska et al. (1998) demonstrated claustrocortical connectivity with area 17 in rat, rabbit, guinea pig and cat. Other studies on rats (Shameem et al., 1984; Sloniewski et al., 1986), cats (Jayaraman and Updyke, 1979; Olson and Graybiel, 1980; LeVay and Sherk, 1981; LeVay, 1986), tree shrews (Carey et al., 1979, 1980), baboons (Riche and Lanoir, 1978) and macaques (Mizuno et al., 1981; Doty, 1983; Kennedy and Bullier, 1985; Perkel et al., 1986) also revealed connections of the claustrum with area 17. Nevertheless, in some studies it is not clear if the projection from the claustrum to area 17 is a result of the injection or through tracer spread to adjacent cortical areas or the underlying white matter (Riche and Lanoir, 1978; Sloniewski et al., 1986). In addition, an interesting observation was made by Carey et al. (1979) in the tree shrew, where they observed that after injection into area 17, retrogradely labeled cells were not seen in the claustrum in every case, but all cases showed labeled cells in the lateral geniculate nucleus. Rather, they observed that retrogradely cells were only found in the claustrum in cases with large injection sites. It seems that the claustrocortical projection to area 17 is quite weak and large injections sites are required in order to visualize this projection. In our study, the injection site was relatively small in order to avoid tracer spread to adjacent areas and the underlying white matter, and this likely resulted in our inability to detect this potential connection. Taken together, the evidence indicates that the claustrum may be connected with area 17; however, this connection is presumably fairly weak and requires large application sites in order to be visualized. It is also possible, given the topographic discontinuities in the representation of the visual field in area 17 of the ferret (Innocenti et al., 2002; Manger et al., 2002a), that our small injections were restricted to a portion of the visual field, for example the peripheral visual field, which does not connect with the claustrum.

### Claustrocortical connectivity to the lower order occipital visual areas 18, 19 and 21

Injection into the visual areas 18, 19 and 21 resulted in a similar overlapping pattern of connectivity, where labeled neurons axon terminals were exclusively found in the caudal third of the ipsilateral claustrum. The rostral extent of the claustral projection zone was highest after injection into area 18, slightly smaller after injection into area 19, and occupied the caudoventral claustrum. The smallest projection zone was observed after injection into area 21, which was limited to the caudal part of the claustrum.

Several studies in the cat (Narkiewicz, 1964; Jayaraman and Updyke, 1979; LeVay and Sherk, 1981; Updyke, 1993; Jakubowska-Sadowska et al., 1998) indicate that the occipital visual areas projection zones in the claustrum are located in the dorsocaudal portion of the claustrum. This is more or less concurrent with the area 21 projection zone in the ferret, where at the most rostral region of connectivity labeled cell were found in the dorsal half of the claustrum. In contrast, visual projection zones of area 18 and 19 were located in the ventral portion of the ferret claustrum, and hence showed an inversion in comparison to the cat. In addition to the claustral connectivity inversion between the cat and the ferret, an inverted retinotopic organization of area 20a, 20b (Manger et al., 2004) and anteromedial lateral suprasylvian visual area (AMLS; Manger et al., 2008) has been observed in the ferret when compared to the cat. Moreover, the ferret retinotopic map within the lateral geniculate nucleus of the thalamus seems to be rotated by approximately 90° in comparison to the cat (Bishop et al., 1968; Zahs and Stryker, 1985), while other visual areas including areas 17, 18, 19, posteromedial lateral suprasylvian visual area (PMLS) and AEV (Manger et al., 2002a, 2005; Cantone et al., 2006) show a similar retinotopy to the cat. Why the retinotopic organization of some of the visual cortical areas and claustral connectivity of ferret differs to those in cat and others not remains uncertain. A possible explanation was provided by Manger et al., 2008, postulating that depending on the developmental time point and maturation of the visual dorsal thalamus and the different cortical areas the retinotopic maps of certain cortical areas are inverted or not (for a detail explanation see Manger et al., 2008). How far this time-dependent developmental concept can be applied to the inverted occipital visual projection zones in the claustrum of ferret is not certain at the moment and requires further investigation.

### Claustrocortical connectivity to the temporal visual areas 20a, 20b and AEV

Areas 20a and 20b are functionally related and adjacent visual areas in the ferret temporal lobe. Nevertheless their claustral labeling showed very different patterns. Injection into area 20b resulted in the labeling of cell and axonal terminals in the caudal third of the ipsilateral claustrum, mostly located in the ventral half. In contrast no projection from the claustrum to area 20a was observed, but area 20a shows an extensive and widespread projection to the claustrum. Anterograde tracing studies with ^3^H-proline in cats demonstrated terminal labeling in the dorsal portion of the claustrum after injection into areas 20a and 20b (LeVay and Sherk, 1981; Updyke, 1993), which agrees with our results after injection in to area 20b in the ferret; however, the terminal field in the claustrum after injection into area 20a in the ferret is more extensive than that seen in the cat. The absence of a claustral projection to area 20a appeared unusual, but this projection has not been analyzed in other animal and thus we cannot exclude that this projection in general exists, it may just be something specific to the ferret. Nevertheless the extensive presence of axon terminals and well-labeled cells following injections into nearby cortical areas makes it very unlikely that the absence of this claustro-area 20a was the result of a methodical problem. Similar to area 20a, AEV seems to project extensively to the entire extent of the contralateral claustrum in the ferret, and it similarly does not receive input from the claustrum. The unidirectional connectivity of areas 20a and AEV, clearly higher order visual areas forming part of the ventral processing stream, is in contrast to the occipital visual areas. It would then appear that these higher order visual areas influence claustral processing, while the claustrum does not directly influence their functionality. What affect this differential connectivity may have on neural processing in these visual areas and the claustrum is presently unknown.

### Claustrocortical connectivity to the parietal visual areas PPc and PPr

PPc and PPr are functionally related visual areas located in the caudal parietal lobe. After injection into areas PPc and PPr labeled cells were found in the dorsal portion of the claustrum. To date a similar connectivity has been demonstrated for the owl monkey (Graham et al., 1979) but not in other species. Area PPc of the ferret seems to have a much broader claustral projection zone than area PPr, which only occupied the caudal third. Nevertheless the projection zone of axonal terminals was more expansive after injection into area PPr than area PPc.

### Connectivity with the contralateral claustrum

No visual cortical area studied herein was observed to receive projections from the contralateral claustrum. Interestingly, only areas PPr and AEV, arguably areas at the terminus of the dorsal and ventral cortical visual processing streams, revealed anterograde projections to the contralateral claustrum. Tracing studies in cats demonstrated that the contralateral claustrum is reciprocally connected to all visual areas studied to date, although these contralateral projections were reported to be far weaker than the ipsilateral ones (Norita, 1977; Squatrito et al., 1980; LeVay and Sherk, 1981; Macchi et al., 1981). It is not clear why in our study we only could visualize the projections from PPr and AEV to the contralateral claustrum; however, since our injection areas were relatively small, in order to avoid tracer spread to adjacent areas, it is possible that given the small amount of tracer it was not sufficient to visualize these rather weak projections. Jakubowska-Sadowska et al. (1998) also failed to visualize the bilateral projection of the claustrum, but they also argue that it is possibly related to small injection sites. Thus, the contralateral corticoclaustral connectivity might be very weak and require large amounts of tracer to be revealed. This indicates that the physiological effect of this connection on neural processing is likely not to be very significant.

### Summary

With the exceptions of areas AEV and 20a, all visual areas that we analyzed appear to be reciprocally connected with the ipsilateral claustrum and the highest density of retrogradely labeled cells were found in the caudal third of the claustrum. Previous studies on different animals (rat, cat, guinea pig, rabbit, Jakubowska-Sadowska et al., 1998; monkey, Graham et al., 1979; cat and baboon, Riche and Lanoir, 1978; cat, LeVay and Sherk, 1981) demonstrated cells projecting to lower order visual cortical areas in the caudal claustrum. Our data reveals that even if the higher order temporal and parietal visual areas have broader projection zones in the claustrum, the ferret caudal claustrum is particularly involved in visual information processing. This finding provides further evidence that the distribution of claustral projection zones along the rostrocaudal axis is phylogenetically stable (Mincinacchi et al., 1985; Jakubowska-Sadowska et al., 1998).

Previous studies have also demonstrated that cortico-claustral connectivity is reciprocal. This was the case for areas 18, 19, 21, PPc and 20b in the ferret, but areas PPr and AEV appear to be an exception to this general pattern of connectivity. Claustral projection neurons to area PPc were found in the caudal half of the claustrum, whereas area PPr seems to project to the entire claustrum. Moreover all higher order visual areas (PPr, PPc, 20a, AEV) studied herein, with the exception of 20b, appear to project, more or less, to the entire claustrum. This broad connectivity with the claustrum may be reflective of the complex types of neuronal information processed in higher order areas. Taken together our results suggest that lower order visual areas reveal more or less clearly defined projection zones located in the caudal claustrum. In comparison to that higher order visual areas, even if not sending and/or receiving projection from the entire claustrum, show a more widespread claustral connectivity rather than a strictly defined projection zone. It would be of interest to compare or combine the results of the connectional studies provided herein with a proteomic redefinition of the claustrum in the ferret as previously done for the rat and monkey (Mathur et al., 2009).

## Conflict of interest statement

The authors declare that the research was conducted in the absence of any commercial or financial relationships that could be construed as a potential conflict of interest.
